# Elective surgery cancellations in pediatric surgery: rate and reasons

**DOI:** 10.1186/s12887-023-04184-x

**Published:** 2023-08-02

**Authors:** Maha Ben Mansour, Oussama Lassioued, Sawsen Chakroun, Amine Slimene, Sabrine Ben Youssef, Amine Ksiaa, Mourad Gahbiche

**Affiliations:** 1 Department of Anesthesiology and Reanimation, Fattouma Bourguiba University Hospital, Monastir, Tunisia; 2 Department of Orthopedics and Traumatology, Fattouma Bourguiba University Hospital, Monastir, Tunisia; 3 Department of Pediatric Surgery, Fattouma Bourguiba University Hospital, Monastir, Tunisia

**Keywords:** Pediatric surgery, Elective surgery, Operating rooms. cancellation

## Abstract

**Introduction:**

Canceling pediatric elective surgery leads to multiple disturbances regarding the inefficient operating room (OR) management, the financial repercussions, and the psychological impact on the patient and his family. This study aims to identify the reasons for cancellations among the pediatric population in our setting and suggest some convenient solutions.

**Methods:**

We carried out a prospective and descriptive study over 12 months in the pediatric surgery department of Fattouma Bourguiba University Hospital.

**Results:**

One thousand four hundred twenty-six patients were scheduled for surgery at the pediatric surgery department, of whom 131 (9.2%) were canceled. Medical and anesthesia-related reasons accounted for 62.5% of all cancellations, followed by surgical reasons at 16%, organizational or administrative issues at 11.5%, and patient-related reasons at 10%. The most significant causes were upper respiratory tract infections (URTIs) in 36.6%, abnormal blood test results in 16%, and non-adherence to preoperative fasting in 9.2%.

**Conclusions:**

The rate of pediatric elective surgery cancellations at Fattouma Bourguiba University Hospital was higher than the accepted average rate (5%). Therefore, to prevent these cancellations as much as possible, efforts should be made to promote children’s medical care, operation scheduling, and efficient institution resource utilization.

## Background

Elective pediatric surgery scheduling is an essential step in treating a child. Therefore, canceling these interventions may lead to numerous disturbances, including patient dissatisfaction, treatment delays, mismanagement of the OR list, and deleterious impacts on young doctors’ training. On this account, the cancellation of pediatric surgeries causes significant emotional trauma and frustration to the child as well as to the parents, who often have to wait months for surgery and may have taken time off of work or traveled long distances only to find out that it had been withdrawn at the last minute [1]. Furthermore, it is well recognized that canceling elective operations significantly affects hospital resources, given the increasing expenses related to prolonged hospital stays and the recurrence of preoperative measurements [2]. Elective surgery cancellations are a universal problem, with reported rates ranging from 1.96 to 24%. This rate varies from one medical structure to another due to the vast heterogeneity of causes [3, 4]. In numerous studies, the most frequent causes of day-of-surgery cancellations are medical issues and work-ups. They highlighted the effectiveness of setting up a preoperative assessment unit where patients complete their preoperative assessments and obtain medical clearance a few days before surgery to avoid these reasons, minimize cancellations and delays, and reduce psychological and economic burdens [4, 5].

Accordingly, this study aims to evaluate the incidence of pediatric surgery cancellations within our teaching hospital by identifying the reasons for postponements and discussing appropriate recommendations to minimize their occurrence.

## Methods

We carried out a prospective and descriptive study over 12 months, from January to December 2022, in the pediatric surgery department of Fattouma Bourguiba University Hospital, which has a 40-bed capacity and an OR theater with three suites. According to the pediatric surgery OR’s functioning, we defined elective surgeries as those planned and scheduled between 8 a.m. and 4 p.m. of the day prior to the surgery date.

For this study, data on elective surgeries were prospectively obtained from the preoperative anesthetic consultation paper, which contains the following information: the demographics of the patient (the ID number or name, the age, and the gender), the intended procedure, and the physical status classification system of the American Society of Anesthesiologists (ASA), as well as the reasons for cancellations, which were classified into the following four predefined groups: Anesthetic or medical reasons (e.g., incomplete preoperative evaluation or treatment), surgical causes (e.g., unavailability of the surgeon or compatible blood supply), organizational or administration-related issues (e.g., OR inaccessibility, insufficient supplies, or lack of personnel), and reasons associated with patients (e.g., non-adherence to fasting).

As part of this study, we restricted the cancellation analysis to patients under 15 and surgeries canceled on the day of surgery. In addition, patients admitted for the resumption of an operative procedure done the previous day, emergency and life-saving surgeries, and minor ambulatory surgeries performed outside the OR unit were excluded.

The cancellation’s causes were further divided into avoidable/foreseeable and unavoidable/ unforeseeable reasons. The latter were defined as those that would not have happened if the patient’s medical records had been well reviewed or if there had been better communication among hospital staff before the day of surgery. Identifying these avoidable cancellations will potentially help sort out practical solutions.

## Results

A total of 1426 pediatric surgeries were scheduled to undergo elective procedures during the study period, of which 131 were canceled on the day of surgery for various reasons. The latter resulted in a mean cancellation rate of 9.2%.

Of the total number of patients whose operation was canceled, 122 (93.1%) experienced one cancellation, and 9 (6.9%) patients had their procedure twice postponed, among whom 6 cases were presented on the day of surgery with non-healed upper respiratory tract infections (URTIs), 2 cases were due to blood group phenotype unavailability, and 1 case was because of the non-existence of a postoperative intensive care unit bed.

The scheduled patients’ demographic characteristics are illustrated in Table 1. The average age of all included patients was 4.27 years ± 1.76 SD. However, the age of those who had their surgery canceled was 3.6 years ± 2.42 SD, with a minimum of 18 days and a maximum of 11 years. The age group from 1 month to 2 years represented 40.5%, with a male predominance of 70.2%. The sex ratio was 2.35.

**Table 1:** Demographic characteristics of scheduled patients

Demographic characteristics	Success group	Canceled group		Total
**Age n (%)**			**Total (%)**	**n (%)**
< or = to 1 month	78 (6%)	6 (4.6%)	**7.1%**	84 (6%)
[1 month -2 years]	423 (32.7%)	53 (40.5%)	**11.1%**	476(33.4%)
[2 years- 5 years]	545 (42.1%)	43 (32.8%)	**7.3%**	588(41.2%)
> 5 years	249 (19.2%)	29 (22.1%)	**10.4%**	278(19.5%)
**Gender**		n (%)	**Total (%)**	
Male	872 (67.3%)	92 (70.2%)	**9.5%**	964(67.6%)
Female	423 (32.7%)	39 (29.8%)	**8.4%**	462(32.4%)
**ASA score**		n (%)	**Total (%)**	
ASA I	987 (76.2%)	101 (77.1%)	**9.2%**	1088(76.3%)
ASA II	211 (16.3%)	17 (13%)	**7.4%**	228 (16%)
ASA III	97 (7.5%)	13 (9.9%)	**11.8%**	110 (7.7%)
**Surgical Specialties**		n (%)	**Total (%)**	
Abdominal surgery	711 (54.9%)	89 (67.9%)	**11.1%**	800 (56%)
Oncology surgery	189 (14.6%)	13 (9.9%)	**6.4%**	202(14.2%)
Cardiothoracic surgery	94 (7.3%)	10 (7.7%)	**9.6%**	104 (7.3%)
Proctology	140 (10.8%)	10 (7.6%)	**6.6%**	150 (10.5%)
Urology	161(12.4%)	9 (6.9%)	**5.3%**	170 (12%)
**Type of surgery**		n (%)	**Total (%)**	
Outpatient surgery	473(36.5%)	52 (39.7%)	**10%**	525(36.8%)
Inpatient surgery	822(63.5%)	79 (60.3%)	**8.7%**	901 (63.2%)

*ASA* American Society of Anesthesiologists physical status classification

In 77.1% of cases, the vast majority of canceled patients were classified as ASA I. However, patients who showed ASA III physical status were annulled in 11.8% of cases, followed by the ASA I group in 9.3% and the ASA II group in 7.4%.

Canceled procedures were divided into different surgical subspecialties. The highest rate occurred in abdominal surgery (67.9%), followed by oncology surgery (9.9%). Of all the surgeries that had to be canceled, 17.6% were for inpatients, while 82.4% were for outpatients.

The most common category for cancellation was attributed to anesthetic or medical reasons, which occurred in 62.5% of cases. URTIs are the most common cause in this group, accounting for 36.6% of this rate 17% are associated with suspicion of a COVID-19 infection. Abnormal blood tests were mentioned in 16% of cases. Sixteen percent of cancellations were due to surgical reasons, with a lack of blood supply (5.3%) being the central issue in this category.

**Fig. 1:**
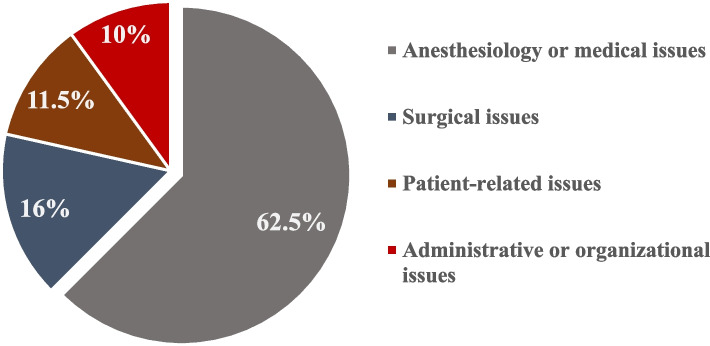
Distribution of categorical reasons for postponed surgeries

Organizational or administrative issues were noticed in 11.5% (*n* = 15), and of these, OR unavailability (*n* = 9) due to technical difficulties or overrunning of previous surgeries accounted for the most cancellations. The remaining 10% (*n* = 13) was attributed to patient-related factors, with 12 cases (92%) being canceled due to fasting nonobservance (see Fig. 1 and Table 2).

**Table 2:** Detailed surgical cancellations according to categorical reasons

Categorical reasons	n	%
**Anesthesiology or medical issues**	**82**	**62.5**
Upper respiratory tract infections (URTIs)	48	36.6
Abnormal test	21	16
Decompensation of preexisting chronic disease	5	3.8
Incomplete preoperative evaluation	5	3.8
Difficult intubation	2	1.5
Contraindication to anesthesia	1	0.8
**Surgical issues**	**21**	**16**
Non-availability of blood	7	5.3
Change in therapeutic attitude	5	3.8
Unavailable surgeon	5	3.8
Rectification of the diagnosis	3	2.3
Missing /defective surgical material	1	0.8
**Patient-related issues**	**13**	**10**
Patient not fasting	12	9.2
The patient’s family refused surgery/patient did not show up for surgery	1	0.8
**Administrative or organizational issues**	**15**	**11.5**
Unavailable operating room	9	6.9
Unavailability of intensive care bed	3	2.3
Staff unavailable	2	1.5
Admission papers not ready	1	0.8

The details of the reasons for the cancellation of elective surgeries are exposed in Table 2.

**Fig. 2:**
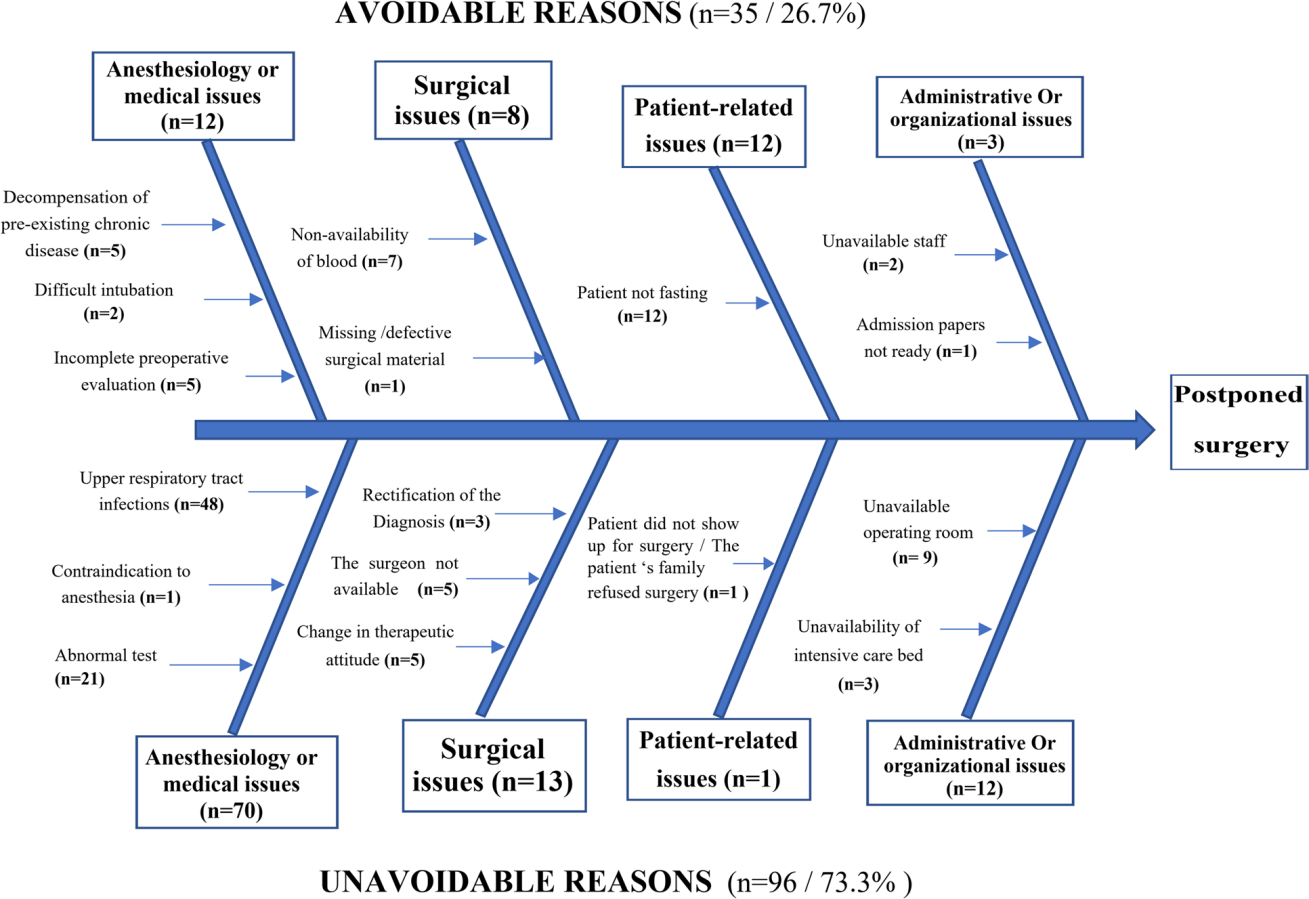
Distribution of cancellation’s causes (avoidable versus unavoidable)

As shown in Fig. 2, the most significant number of cancellations were deemed unavoidable (*n* = 96, 73.3%). In comparison, 35 cases (26.7%) were judged as potentially avoidable. In this category, most cancellations were related to the non-respect of the fasting period (*n* = 12), the lack of compatible blood supplies (*n* = 7), incomplete preoperative evaluation (*n* = 5), and decompensation of preexisting chronic disease (*n* = 5). The majority of the unavoidable cancellations were due to URTIs (*n* = 48), unexpected abnormal tests (*n* = 21), and operating room unavailability (*n* = 9).

At the end of the study period, 75 (56.9%) patients were rescheduled and operated on within seven days. The remaining 56 patients (43.1%) were discharged, and surgery was postponed for at least a month (Table 3).

**Table 3:** Delays in scheduling surgical procedures

Elective surgery delay	n	%
Within 48 h	26	19.5
Within one week	49	37.4
Within one month	24	18.3
After one month	32	24.4

## Discussion

While no consensus has been reached on the acceptable cancellation rate, recent studies have established a limit of 5% that institutions should not exceed to prevent the waste of resources [4]. In the current study, we discovered a 9.2% cancellation rate, which appears reasonable compared to Algeria’s 13.7% [6], Nigeria’s 28.5% [5], and the Caribbean’s 30.1% rates [7]. However, compared to developed countries, where rates range from 1.97% in France [8] to 6.5% in Spain [9] and 7.2% in Australia [10], our percentage is higher and needs measurements to be improved. The differences in results among studies can also be attributed to the following reasons:

- The time definition for elective surgeries
- The presence of multiple classifications of cancellation reasons
- The setting of the surgical department and the hospital (community hospital versus teaching hospital)
- The difference in surgical subspecialty requirements and type of surgery (inpatient versus outpatient)
- The number of included patients
- The period of the study
- The attitude regarding emergency surgeries

Based on our findings, we noted an average age of 3.6 years, under the age of the scheduled population, and a high proportion of infants aged one month to two years whose surgeries were canceled. The latter could be explained by the fact that younger patients have less tolerance for preoperative fasting and are more susceptible to URTIs, which generally result in a significant proportion of cancellations [6, 7].

Our study revealed that males had a cancellation rate of 9.5%, higher than 8.4% among females. This finding contradicts the results of the Sudan study [2], which showed higher female cancellation rates. Unfortunately, we could not determine the reasons for these gender differences.

Additionally, we found that 77.1% of canceled patients were classified as ASA I. However, the ASA III patients had the highest cancellation rate at 11.8%. This is quite comparable to Benouaz et al. [6], who reported a higher rate of cancellations among ASA III patients (20%), mainly explained by this group’s higher medical co-morbidities. Besides, it is worth noting that our patients are also canceled for reasons other than their physical fitness status, given that cancellations in our department are ASA Grade I in most cases.

Abdominal surgery had the highest number of elective pediatric surgery cancellations, while oncology and urology had the lowest. The variability between surgical specialties may be explained by the fact that our series mainly consists of visceral procedures. These results are not comparable to the Benouaz study [6], where the cancellation was more related to urologic and orthopedic surgeries.

It was observed that the rate of cancellations among inpatients (8.7%) was lower compared to outpatients (10%), which aligns with the findings of Gonzalez-Arevalo et al. [9]. This disparity may be attributed to our practice of admitting inpatients to the hospital a day before their scheduled operation to ensure better preparation.

The analysis of cancellations showed that the most frequently documented cause was related to anesthetic or medical reasons (62.5%), with URTIs being the most common cause in this category, accounting for 36.6%. This finding is consistent with Bathla et al. [11], who revealed that URTIs were involved in 30.7% of revoked operations.

Due to some elective surgery requirements, the proportion of patients scheduled in the winter is higher than in the summer [6]. Regardless, the risk of these children catching an UTRI is higher in the winter, leading to an increased cancellation rate. The COVID-19 pandemic has contributed to 17% of cancellations for an URTI in our study. However, Skarsgard et al. [12] reported a higher COVID cancellation rate (84%). Therefore, investigation and prevention of airway infections remain essential for the proper conduct of anesthesia to avoid increased risks of respiratory complications, including bronchospasm, laryngospasm, desaturation, and respiratory pauses [13].

According to Moret et al. [14], The abnormal blood test values on the day of presentation (e.g., unexpected hemoglobin level, high white count, high C-reactive protein) resulted in 10.5% of cancellations. However, our result is higher, at around 16%.

Cancellations for surgery-related reasons were the second most common (16%) in the present study. The majority were due to a lack of compatible blood supplies, strongly affected by the COVID-19 pandemic, which limited blood donation. Cancellations could be directly linked to the surgeon in many situations, such as unavailability (illness or travel), change in the treatment plan, experience, and underestimation of the needed time for the operation [4]. Only one of the patients we analyzed was canceled due to a COVID-19 surgeon infection.

According to our study, patient-related reasons were observed in 10% of cases. Similar findings have been reported in the literature: a teaching hospital in Hong Kong reported a 10% cancellation of the elective day of surgery due to the Patient himself [15]. Our results in this category are surprising, considering that non-adherence to fasting is the most common reason (12 cases out of 13).

Current literature also emphasizes the importance of patients’ socioeconomic factors in adherence to operative programs and medical services. This occurs when the patient does not have insurance approval, cannot pay, or the admissions staff has not completed the financial clearance process, and they remain on the final list of elective surgeries in the OR [16].

Administrative or organizational causes of cancellation accounted for 11.5% of cases, explained by the lack of operating theater space, facilities (beds, implants, etc.), and valuable human resources, which are common problems in developing nations’ hospitals. In our resource-limited setting, where staff shortages are a challenging problem, re-distribution of the few staff members available is usually needed to address this issue. The result is better than those of developing countries: Tanzania at 82% [2], Burkina Faso at 70% [17], and Algeria at 41.5% [6]. Nevertheless, it is still lower than in developed countries [14].

Our study’s analysis of the predictability of cancellations showed that 26.7% of cases are potentially evitable by implementing additional precautions. As mentioned, these avoidable causes were mainly linked to fasting period non-respect (34.3%). Our result differs from other series, which had a lower cancellation rate for noncompliance with preoperative fasting. The studies of Benouaz et al. [6], Mesmar et al. [18], and Haana et al. [10] had a rate of 3.7%, 2.6%, and 3.5%, respectively. We attribute this to the fact that the patient is not appropriately instructed to respect fasting, inadequate information transmission between healthcare teams, particularly during shifts, and sometimes to the parents, who may feed their child when waiting time is prolonged.

Then, we firmly believe that only encouraging patients to respect the fasting period and adjusting operation waiting times to align with the patient’s ability to fast can significantly improve our cancellation rate.

Considering the non-avoidable cancellations in our study, they were primarily due to URTIs (50%), which are one of the most controversial issues in pediatric anesthesia, and opinions are divided on whether to take up a patient with URTI for elective surgery, especially as it has been identified as a frequent reason for cancellation in several studies [7, 11, 19]. That is why they recommend asking parents to promptly contact the hospital at the onset of any acute illness [6].

To establish the OR elective surgeries list, our department routinely executes the following procedure: After examining the patient and deciding on the surgery, the pediatric surgeon refers the patient for a pre-anesthesia appointment. The medical secretary will prescribe the needed assessments and exams before the consultation according to the patient’s disease, type of surgery, and ASA classification. The appointment for this consultation will be chosen depending on the surgery date and the patient’s medical status. A patient with severe medical conditions will have a pre-anesthesia consultation closer to the surgery date, possibly with exceptional follow-up during this period. If special preparation is required, scheduling a phone call and an earlier hospitalization may be necessary. However, our method is still criticized for lacking a pre-established explicit policy outlining preoperative assessments. Furthermore, postponing the patient’s assessment until the evening before surgery is sometimes customary, which may lead to more cancellations the next day.

Referring to the literature, we briefly propose specific potential solutions before scheduling patients for elective surgery to prevent or minimize cancellations, especially in cases of avoidable causes:

- Take into consideration the financial status of patients.
- Consider the psychological status of the child and his parents, and try to explain why the operation has been postponed sufficiently.
- OR lists should be realistic, manageable, and take account of material and organizational difficulties [2].
- Ensure a pre-anesthetic consultation and visit before surgery with a sufficient medical exam prescription to detect preventable medical causes [13].
- Ensure the patient and their parent follow preoperative instructions (e.g., fasting) at every stage of the treatment process.
- Enhance team spirit and improve communication between health caregivers (surgeon, anesthesiologist, OR medical director, OR nurse manager, etc.) [20].
- Promote a standardized procedure to have assigned personnel call patients a few days before their surgery date to confirm the respect of the appointment and preoperative instructions and to ask about any acute illness [6, 21]. This method has decreased the postponement rate by 50% in the United States [22] and 83% in Algeria [6]. Some hospitals require a preoperative visit and postoperative follow-up by phone for ambulatory surgeries to unload the hospitalization services and increase the fluidity of turnover, which offers more care possibilities and better satisfaction for the parents [21, 23].
- Prepare feasible alternative arrangements to make in case of an unusual incident and predictive models for last-minute surgery cancellations [24].
- Develop software systems that can facilitate checking tasks [4].
- Prioritize future improvement efforts, principally on avoidable surgical cancellations.

## Limitations

The weakness of this study is related to the small number of included patients. In addition, it took place during the last waves of the COVID-19 pandemic, which may have influenced the results, especially with the medical, material, and logistical shortages during the pandemic.

Furthermore, in some cases, the reasons for cancellations were multifactorial. So, this may have contributed to bias in categorizing cases.

Different definitions and classifications of cancellations in the literature may have been a source of ambiguity when comparing different studies with our own. Finally, the current study was limited to one teaching hospital, so it is difficult to generalize the results to all other medical structures.

## Conclusions

Compared to the recommended rate (5%), the incidence of pediatric elective surgery cancellations on the scheduled day in our study was acceptable (9.1%). However, this percentage can be easily reduced if efforts are made to prevent the most frequent avoidable causes, especially fasting noncompliance. To improve the operating theater efficiency in the pediatric surgery department and to reduce the institution’s financial expenses, we recommend establishing a systematic preoperative consultation a few days prior to surgery, integrating the parents in their child’s care and considering their socioeconomic and psychological status, promoting communication between OR staff members, taking into account the local constraints in terms of human and material resources, and finally leading continuous assessments of the OR activity to detect any dysfunction or unnecessary cancellations early.

## Data Availability

The datasets used and/or analyzed during the current study are available from the corresponding author upon reasonable request.

## Abbreviations

URTIs: Upper respiratory tract infections
ASA: American Society of Anesthesiologists
OR: Operating Room
